# The immunomodulatory effects of endocrine therapy in breast cancer

**DOI:** 10.1186/s13046-020-01788-4

**Published:** 2021-01-07

**Authors:** Huanhuan Huang, Jun Zhou, Hailong Chen, Jiaxin Li, Chao Zhang, Xia Jiang, Chao Ni

**Affiliations:** 1 Department of Breast Surgery, Second Affiliated Hospital Zhejiang University, Zhejiang 310009 Hangzhou, China; 2 Key Laboratory of Tumour Microenvironment and Immune Therapy of Zhejiang Province, Second Affiliated Hospital Zhejiang University, Zhejiang 310009 Hangzhou, China; 3 Department of Breast Surgery, Affiliated Hangzhou First People’s Hospital Zhejiang University, Zhejiang 310006 Hangzhou, China; 4 Department of Anatomy School of Medicine, Zhejiang University, Zhejiang 310058 Hangzhou, China; 5 School of Public Health and West China Fourth Hospital, Sichuan University, Chengdu, Sichuan 610064 China; 6 Department of Clinical Neuroscience Centre for Molecular Medicine, Karolinska Institute, Stockholm, 17176 Sweden

**Keywords:** Breast cancer, Endocrine therapy, PI3K-AKT-mTOR pathway, CDK4/6, Tumor immune microenvironment

## Abstract

Endocrine therapies with SERMs (selective estrogen receptor modulators) or SERDs (selective estrogen receptor downregulators) are standard therapies for patients with estrogen receptor (ER)-positive breast cancer. Multiple small molecule inhibitors targeting the PI3K-AKT-mTOR pathway or CDK4/6 have been developed to be used in combination with anti-estrogen drugs to overcome endocrine resistance. In addition to their direct antitumor effects, accumulating evidence has revealed the tumor immune microenvironment (TIM)-modulating effects of these therapeutic strategies, which have not been properly acknowledged previously. The immune microenvironment of breast tumors plays a crucial role in tumor development, metastasis and treatment response to endocrine therapy and immunotherapy. Therefore, in our current work, we comprehensively review the immunomodulatory effect of endocrine therapy and discuss its potential applications in combination with immune checkpoint inhibitors in breast cancer treatment.

## Background

Breast cancer (BC) remains the most common malignant tumor threatening women’s health worldwide [1]. As more than 75% of the diagnosed cases express estrogen receptor alpha (ERα) [2], it has been considered the most important target of endocrine therapies. Since tamoxifen was first discovered for its antitumor function in BC, several anti-estrogen regimens, including SERMs (selective estrogen receptor modulators), SERDs (selective estrogen receptor downregulators), AIs (aromatase inhibitors) and GnRHa (gonadotropin-releasing hormone antagonists), have been developed to improve patient outcomes [3], yet endocrine resistance and disease progression still occurs in approximately 50% of these patients [4]. Activation of PI3KCA-AKT-mTOR pathway is known as the most important and prevalent mechanism of endocrine resistance in BC, as clinical trials have confirmed the definite efficacy of drugs targeting PI3KCA, AKT or mTOR [5–7]. Moreover, regimens targeting the cell cycle regulatory protein CDK4/6 in combination with anti-estrogen regimens (SERDs/AIs) have also been demonstrated to greatly improve the prognosis of ERα + BC [8, 9]. In addition to the direct antitumor effect of these endocrine therapies and small molecular inhibitors, increasing lines of evidence has highlighted a complex interplay between them and the tumor immune microenvironment (TIM), which is mainly composed of T cells, B cells, dendritic cells, macrophages, neutrophils, etc.. This interplay further affects tumor progression and endocrine resistance in BC [10–12]. However, the immune modulatory effect of the above indicated anti-estrogen regimens and small molecule inhibitors has rarely been systematically discussed. Therefore, we aim to provide a comprehensive review on the impact of various endocrine therapeutic strategies on the TIM in BC covering SERDs, SERMs, AIs, GnRHa and inhibitors of PI3K, AKT, mTOR and CDK4/6.

## The effects of estrogen on the immune system

Estrogen, an aromatized steroid hormone produced mainly in gonads and extraglandular tissues, participates in a wide range of physiological processes through the ER signaling pathway. Although the production of estrogen by ovaries ceases among postmenopausal women, estrogen is continually generated by the adipose tissue [13], of which both normal and tumor-bearing breasts are enriched. It has been estimated that the level of estradiol in breast tumor is 50–100 fold higher than that in normal breast tissue or in circulation, indicating an *in situ* synthesis capacity of breast tumor cells [14]. Indeed, acquired *CYP19A1* (encoding aromatase) amplification in BC cells has been found in 21.5% of relapsed AI-treated patients, and this alteration causes autonomous estrogen biosynthesis that activates ERα [15]. Moreover, the extensive expression of ERα/β in immune cells further supports the abnormal regulatory action of estrogen on immune system elements involving their development and functional responses [16].

Multiple studies have highlighted a pleiotropic effect of estrogen on immune cells involving polarization, cytokines production, proliferation and effector function. Macrophages, as part of the innate immune system, are one of the predominant infiltrating immune cells associated with breast tumor progression [17]. Estrogen can preferentially induce alternative activated (M2) macrophages which usually sustain tumor progression [18]. Multiple cytokines produced by functional macrophages, including matrix metalloproteinase-9 (MMP-9), IL-6, TNFα and IL-1β, are dampened under the action of estrogen-ERα signaling [19–21], exerting both antitumor and protumor functions. Estrogen is known to directly or indirectly inhibit NK cell cytotoxic activity, the latter function of which depends on estrogen-induced production of the granzyme B inhibitor protease inhibitor 9 (PI-9) by target cells, including BC cells [22, 23]. Furthermore, the effect of estrogen on DCs varies at different stages. On the one hand, estrogen promotes the differentiation of DC progenitors and costimulatory molecule expression on differentiated conventional DCs [16, 24]. On the other hand, antigen presentation by mature DCs can be impaired after estrogen treatment, with decreased secretion of IFNγ, TNFα and IL-12 [25]. The increased production of indoleamine 2,3-dioxygenase (IDO) in DCs induced by estrogen reveals its suppressive function on antigen-specific T cells [26]. Estrogen has also been shown to directly inhibit the proliferation of CD4 + T cells as well as to reduce the expression of IL-2 and IL-2R [27]. In addition, estrogen also promotes the amplification and immunosuppressive capacity of regulatory T cells (Tregs) and myeloid-derived suppressor cells (MDSCs) [28, 29]. The expression of Foxp3 and programmed cell death 1 receptor (PD-1) responsible for the suppressive function of Tregs can be upregulated by estrogen in an ERα-dependent manner [28, 30]. Estrogen further induces the infiltration and activation of neutrophils with an increased expression of protumoral cytokines and chemokines (e.g. S100A-8, S100A-9, CXCL-1, and CXCL-2), tissue-remodeling enzymes (MMP-3 and MMP-9) and COX-2 during mammary involution [31]. Protumoral neutrophils exacerbate the formation of tumor-promoting microenvironment, which further encourages BC progression [31, 32].

**Fig. 1:**
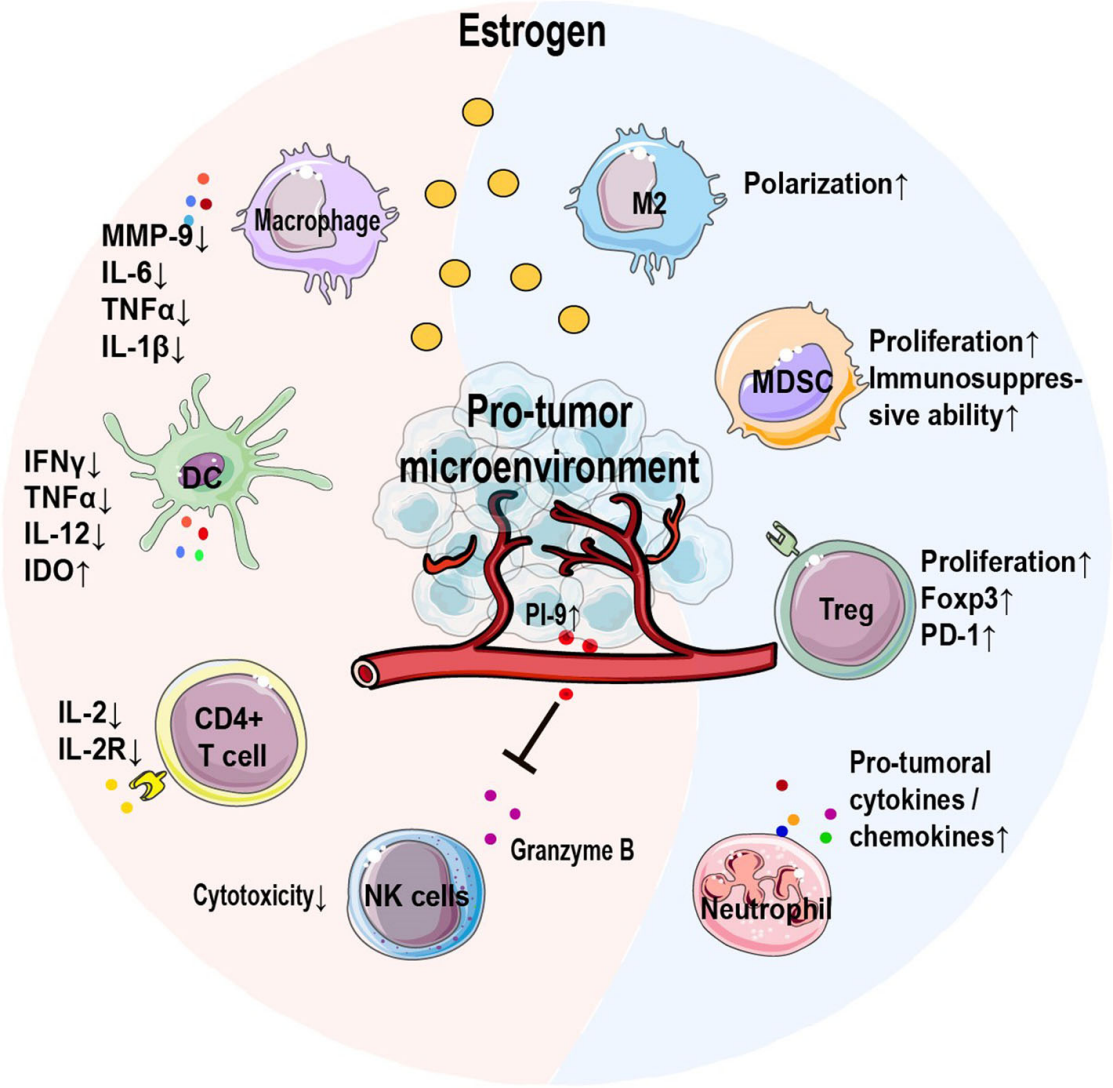
Estrogen induces suppressive tumor immune microenvironment. Estrogen predominantly plays an immune-suppressive function in the TIM. Estrogen reduces multiple cytokines in macrophages and preferentially polarizes macrophages towards the M2 subtype. The infiltration of neutrophils with a protumoral signature may also be induced by estrogen. Both the proliferation and immunosuppressive function of Tregs and MDSCs can be potentiated by estrogen. Estrogen also inhibits the function of cytotoxic T cells, DCs and NK cells through multiple mechanisms

For a detailed description, please also read a review written by Segovia-Mendoza et al. [18] on the immune modulatory effect of estrogen in different immune cells. To summarize as illustrated in Fig. 1, estrogen acts as an immune-suppressive factor in favor of tumor emergence and progression. As such, along with its direct killing effect on cancer cells, anti-estrogen therapy may orchestrate an antitumor/pro-immune surveillance TIM in BC.

## The effects of SERMs and SERDs on the tumor immune microenvironment

SERMs and SERDs are currently the most important endocrine therapeutic regimens for BC. SERMs, represented by tamoxifen, toremifene and raloxifene, work through competitive blockage of the interaction between estrogen and ERα. SERDs, such as fulvestrant, contribute to the downregulation and degradation of ERα [33]. Accumulating evidence from experimental and clinical studies has revealed the multifaceted immunomodulatory effects of SERMs and SERDs [34]; in particular, progress has been made to elaborate how SERMs and SERDs act upon the immune microenvironment of BC (see Table 1).

**Table 1:** The effects of different endocrine therapeutic strategies on immune cells

Therapeutic strategy	Subdivision	Drug/inhibitor	Modulate TIM component	Effects on immune cells
Anti-estrogenic drugs	Selective estrogen receptor Modulators (SERMs)	tamoxifen, toremifene, raloxifene	CD8+T cells	proliferation↑ [35], cytotoxicity↓ [12]
			CD4+T cells	Treg polarization↑ [12]
			NK cells	cytotoxicity↑ [36]
			DCs	functional differentiation and immunostimulatory capacity↓ [37]
			neutrophils	tamoxifen improves the proinflammatory pathway [38], while raloxifene has the opposite effect [39]
	Selective estrogen receptor down-regulators (SERDs)	fulvestrant	CD8+T cells	cytotoxicity↓ [12], tumor infiltration↑ [40]
			CD4+T cells	Treg polarization↑ [12], tumor infiltration↑ [40]
			MDSCs, Tregs	tumor infiltration↓ [40]
			DCs	tumor infiltration↑ [40]
	Aromatase inhibitors (AIs)	letrozole, anastrozole, exemestane, formestane	CD8+T cells	tumor infiltration↑ [41]
			CD4+T cells	Treg polarization↓ [42]
			Tregs	ERβ inactivation induces immunosuppressive activity↓ [43]
			mast cells	ERβ inactivation induces CCL-2 production↓ [44]
	Gonadotropin-releasing hormone antagonists (GnRHa)	Goserelin triptorelin	T cells	induce TH1 shift [45]
Inhibition of the PI3K-AKT-mTOR pathway	PI3K inhibitors	pan-PI3K inhibitor	macrophages	proinflammatory cytokines production and motility↓ [46]
			Tregs	proliferation↓ [47]
			T cells	cytokines and granzyme B secretion↓ [48], tumor infiltration↑ [49]
			NK and B cells	tumor infiltration↑ [49]
		p110α inhibitor	CD8+ T cells	tumor infiltration↑ [50], cytokines production and cytotoxicity↑ [51],
			CD4+ T cells	cytokines production↑ [51], Treg polarization↓ [51]
			MDSCs	tumor infiltration↓ [52]
		p110β inhibitor	macrophages	phagocytosis↓ [53]
			neutrophils	cell adhesion, spreading and ROS formation↓ [54]
		p110γ inhibitor	myeloid cells	tumor infiltration↓ [55]
			macrophages	M1 polarization↑ [56]
			CD8+T cells	PD-1 and CTLA-4 expression↑ [57], tumor infiltration↑ [49]
			CD4+Tcells, B cells	tumor infiltration↑ [49]
		p110δ inhibitor	macrophages	tumor infiltration↓ [58]
			MDSCs, Tregs	immunosuppressive function↓ [59]
			T cells	effector response of effector/memory T cells↓ [60]
			B cells	proliferation, survival and differentiation↓ [61]
	AKT inhibitors	capivasertib	Tregs	proliferation↓ [47]
			MDSCs	differentiation and viability↓ [62]
			macrophages	AKT1 ablation →M1 phenotype, AKT2 ablation →M2 phenotype [63]
	mTOR inhibitors	rapamycin, everolimus	mononuclear cells	polarization towards M1 macrophage
			macrophages	proinflammatory cytokine production and motility↓ [46]
			NK cells	proliferation and cytotoxicity↓ [64, 65]
			DCs	CD40, CD86↑, PD-L1↓ [66]
			CD8+ T cells	anergic state induction [67, 68], tumor infiltration↓ [69]
			CD4+ T cells	induce Tregs polarization and Foxp3 stable expression [70]
			γδT cells	cytotoxicity of Vγ4γδT cells↑ [71], proliferation and cytotoxicity of Vγ2Vδ2 T cells↑and apoptosis↓ [72]
			Tregs	transient mTOR inhibition: reverse the hyporesponsiveness [73] chronic mTOR inhibition: proliferation↓, suppressive function↓ [73, 74]
Inhibition of the cell cycle	CDK4/6 inhibitors	abemaciclib, palbociclib, ribociclib	myeloid cells	tumor infiltration↓ [75]
			macrophages, DCs	antigen presentation↑ [76]
			T cells	PD-1 and CTLA-4 expression↑ [52], activation↑, IL-2 production↑ [75]
			Tregs	inhibition of the cell cycle [77]

In a mouse BC model with tamoxifen resistance, genes relevant to the immune system processing were found to be upregulated. Subsequent gene ontology analysis showed that a majority of upregulated genes were associated with interferon pathways, with interferon regulatory factor 7 (IRF-7) identified as a key regulator of the downstream IFN-dependent immune responses linked to tamoxifen resistance [78]. An immune-polarizing side effect (IPSE) of tamoxifen has also been reported. Tamoxifen induced a shift of mouse myelin-specific CD4 + T cells from a TH1 phenotype targeted against tumor cells towards a TH2 phenotype, indicating its damaging effect on antitumor immunity [34, 79]. Consistent with experimental data, patients with low tumor-induced TH2 polarization status before treatment showed better prognosis due to their better resistance against the IPSE of tamoxifen [80]. In addition, Joffroy et al. [12] found that tamoxifen- or fulvestrant-induced TGFβ production in MCF-7 cells led to a decreased cytotoxic effect of CD8 + T cells as well as an increased polarization of CD4 + T cells into Foxp3 + Tregs, which futher supported the development of endocrine resistance. Moreover, ERα signaling blockade or depletion by tamoxifen or fulvestrant evoked the upregulation of programmed death-ligand 1 (PD-L1) in multiple ER + BC cell lines, contributing to the cytotoxic T cell evasion of BC cells [81]. The inverse correlation between ERα and PD-L1 was confirmed not only in the MMTV-PyMT transgenic mouse model [81] but also in human BC specimens, where the ratio of PD-L1 positive patients was much lower in ER+/HER2− BC (19.4%) than in triple-negative breast cancer (TNBC, 58.6%) [82, 83]. To summarize, SERMs or SERDs mediate immunosuppressive effects in a direct or indirect manner and protect tumors from immune surveillance, which may decrease their therapeutic effects and result in the development of treatment resistance.

On the other hand, SERMs and SERDs have also been found to enhance the immunogenicity of BC and improve the antitumor immunity. The lactation protein α-lactalbumin, known as an immunotherapeutic target of breast tumor treatment, is usually downregulated during cancer development. Jaini et al. found tamoxifen and fulvestrant effectively promoted α-lactalbumin expression in BC cells while leaving the normal breast tissue unaffected, resulting in enhanced breast tumor inhibition via targeted immune therapy [84]. Furthermore, the cytotoxicity of NK cells was also found to be boosted by tamoxifen, which upregulated c-erbB-2 expression in HER2/neu nonamplified BC and led to tumor cell lysis by NK cell-mediated antibody-dependent cytotoxicity (ADCC). Interestingly, in cells presenting HER2/neu amplification, although tamoxifen upregulated HER2/neu, it failed to improve sensitivity to the cytotoxic effect of NK cells. This may be explained by a ceiling effect induced by the limited Fc receptors on NK cells [36]. Similar to tamoxifen, toremifene upregulates intercellular adhesion molecule-1 (ICAM-1) expression on MCF-7 cells, and ICAM-1 is a key factor bridging and forming the immunological synapses between NK and target cells [85]. Moreover, estrogen-induced tumor immune tolerance was revealed to be partially abolished by tamoxifen though suppression of FasL expression as well as blockade of cancer-derived CCL-2/CCL-5 [86, 87]. Intriguingly, SERDs (either fulvestrant or JD128), despite lacking a direct antitumor effect on ER-negative BC, were found to reduce the counts of MDSCs and Tregs as well as increase the infiltration of DCs and CD8 + and CD4 + T cells in 4T1 tumor-bearing mice. These changes in immune cell subpopulations in the TIM elicited by SERDs significantly improved the efficiency of anti-PD-L1 therapy [40]. Beyond SERMs/SERDs-induced antitumor immunity, the upregulated expression of PD-L1 on tumor cells also raises the potential of using anti-estrogens in combination with immune checkpoint blockers (ICBs) in BC. A multitude of clinical trials (see Table 2) evaluating the efficiency of ICBs in combination with anti-estrogen therapy are ongoing and will release their findings in the near future.

**Table 2:** Ongoing clinical trials of endocrine therapy combined with immune checkpoint inhibitors therapies for breast cancer

Treatment arms	Clinicaltrials.gov identifier	Phase	Patient and enrollment criteria	Primary Outcome Measures	Secondary Outcome Measures	Completion Date
Pembrolizumab + Tamoxifen	NCT03879174	2	Advanced hormone receptor- positive breast cancer and ESR1 mutation	PFS, ORR	OS	August 1, 2022
Pembrolizumab + Fulvestrant	NCT03393845	2	Hormone receptor-positive, HER2-negative advanced/metastatic breast cancer	ORR	Safety profile	January 1, 2022
Pembrolizumab + Exemestane + Leuprolide	NCT02990845	1/2	Premenopausal hormone receptor positive/HER2 negative locally advanced or metastatic breast cancer	PFS	AEs, ORR, CBR, DOR	December 2021
Durvalumab + Aromatase Inhibitor (Anastrozole/Letrozole/Exemestane)	NCT03874325	2	Hormone receptor positive breast cancer	Rate of modified preoperative endocrine prognostic index (mPEPI) score of 0	CR, PR, PD, SD	March 11, 2025
Atezolizumab + Fulvestrant, Atezolizumab + Ipatasertib, Atezolizumab + Ipatasertib + Fulvestrant	NCT03280563	1/2	Hormone receptor positive, human epidermal growth factor receptor 2-negative breast cancer	OR	PFS, CBR, OS, DR, AEs	October 5, 2022
Pembrolizumab + Letrozole + Palbociclib	NCT02778685	2	Estrogen receptor positive, HER2/neu negative, postmenopausal metastatic breast carcinoma, stage IV breast cancer	CR, PR	CRR, DOR, AEs, OS, PFS, TTF	September 2020^a^
Fulvestrant + Palbociclib + Avelumab	NCT03147287	2	Metastatic hormone receptor positive, HER2 negative breast cancer	PFS	ORR, AEs	December 31, 2024
Abemaciclib + Pembrolizumab, Abemaciclib + Pembrolizumab + Anastrozole	NCT02779751	1b	Hormone receptor-positive, HER2-negative breast cancer	SAEs, AEs	ORR, DCR, DOR, PFS, OS, PK	October 29, 2021
Abemaciclib + Durvalumab + Aromatase inhibitor (exemestane/anastrozole/ letrozole)	NCT04088032	1	Locally advanced hormone receptor-positive breast cancer	AEs	Pathologic response at surgery	December 31, 2020
Nivolumab + Palbociclib + Anastrozole	NCT04075604	2	Hormone receptor positive, HER2-negative breast cancer	DLT, RCB,	AEs, SAE, laboratory abnormalities, pCR, ORR, BCS rate	March 9, 2022

Abbreviations: *PFS* Progression Free Survival, *ORR* Overall Response Rate, *OS* Overall Survival, *AEs* Adverse Events, *CBR* Clinical Benefit Rate, *DOR* Duration of Overall Response, *CR* Complete Response, *PR* Partial Response, *PD* Progression of Disease, *SD* Stable Disease, *OR* Objective Response, *DR* Duration of Response, *CRR* Complete Response Rate, *TTF* Time to treatment failure, *SAEs* Serious Adverse Events, *DCR* Disease Control Rate, *PK* Pharmacokinetics, *pCR* Pathological Complete Response, *BCS* Breast Conserving Surgery

^a^The clinical study (NCT02778685) is recruiting volunteers, with its estimated completion time to be determined

In addition to being effective for treating BC, tamoxifen and raloxifene have also been shown to reduce the risk of BC in highly susceptible women [88]. Tamoxifen administration was found to upregulate IFN-related genes in normal human mammary epithelial cells from in vitro experiments, implying its positive effect on the immune surveillance of normal breast tissue [89]. Moreover, tamoxifen and toremifene were also found to enhance TNF-R2 expression on activated T cells by inhibiting the activation of JNK and promoting TNF-R2-mediated T cell proliferation [35]. These results might explain their BC-preventive effect.

In contrast, the functional differentiation and immunostimulatory capacity of DCs were affected by tamoxifen and raloxifene, which acted to maintain the immature state of DCs by depressing their response to inflammatory stimuli [37]. Neutrophils, the major effector immune cells in inflammation, were also affected by SERM treatment. Corriden et al. [38] revealed a positive effect of tamoxifen on the proinflammatory processes of human neutrophils, including chemotaxis, phagocytosis and ceramide/PKCζ-mediated neutrophil extracellular traps (NETs) formation. Nevertheless, the same group later found raloxifene exerted an inhibitory effect on ceramide expression in neutrophils and phorbol 12-myristate 13-acetate (PMA)-induced NETs, suppressing the NETs-based killing function of neutrophils against bacterial pathogens [39]. The distinct effects of tamoxifen and raloxifene on NETs formation in neutrophils might be due to their slight differences in molecular structure. Since the neutrophil accumulation and NETs formation induced by inflammation are the main processes awakening dormant tumor cells, which can remain quiescent for decades before relapse [90], it is worthwhile to evaluate the effect of tamoxifen and raloxifene on quiescent BC cells, such as cancer stem cells, in preclinical models of BC.

## The effects of estrogen deprivation on the immune microenvironment

**Fig. 2:**
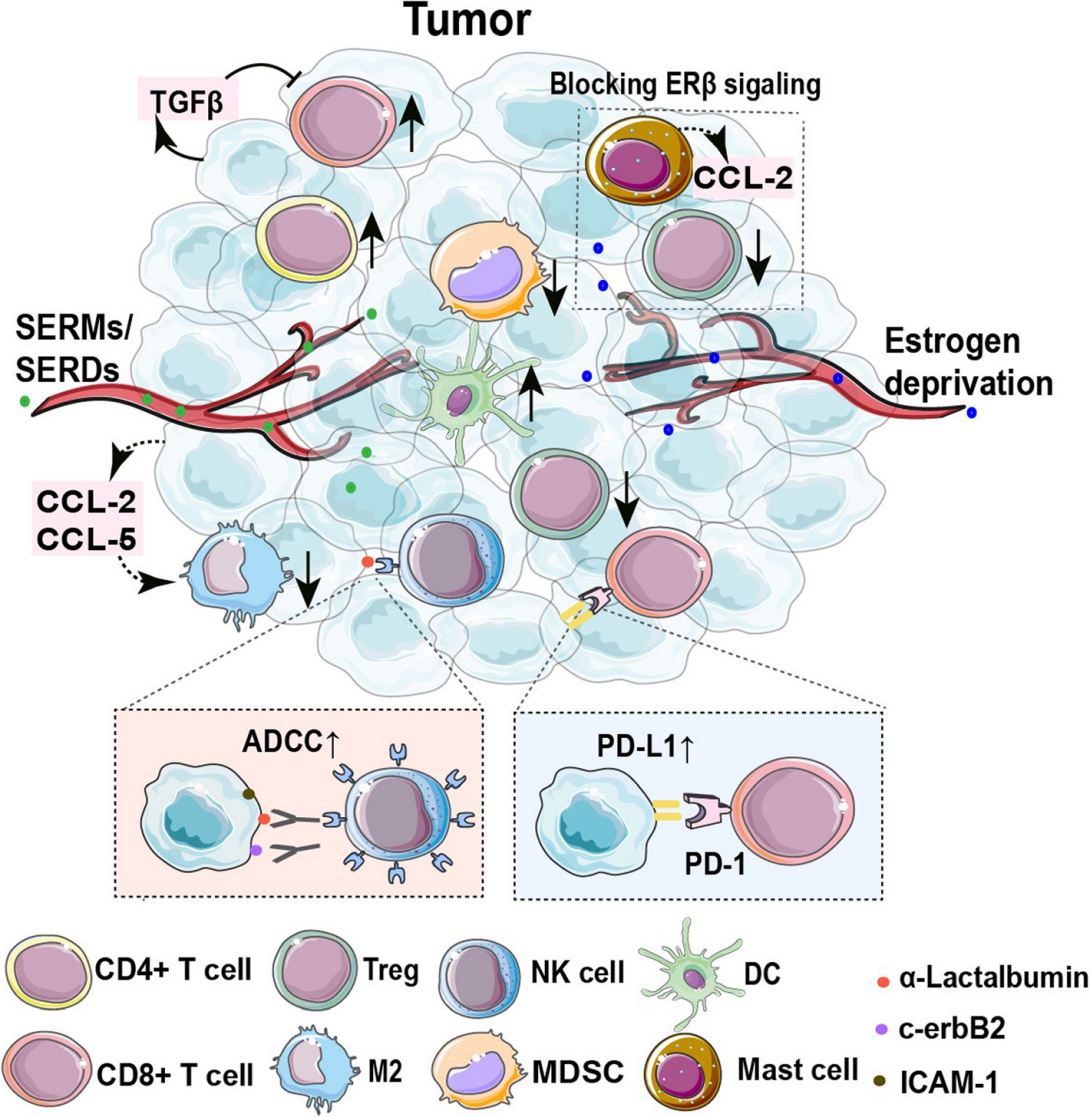
Antiestrogen regimens participate in reshaping tumor immune microenvironment. Blocking or downregulating ERα with SERMs/SERDs and estrogen deprivation can enhance PD-L1 expression and immunogenicity of BC, as well as regulate multiple factors secreted by BC cells, such as TGFβ, CCL-2 and CCL-5, indirectly modulating the tumor immune microenvironment. Enhanced NK cell-mediated antibody-dependent cytotoxicity (ADCC) increased the infiltration of CD4+ T cells, CD8+ T cells, and DCs and decreased the counts of Tregs and MDSCs within tumors can be found after anti-estrogen regimens treatment. Estrogen deprivation can lead to a greater inhibition of Tregs infiltration in tumor and block the production of CCL-2 by mast cells due to simultaneous suppression of ERβ signaling

Estrogen deprivation has been proved to be more effective in postmenopausal or high-risk premenopausal patients treated with either aromatase inhibitors (AIs) alone or AIs combined with a GnRHa (e.g., goserelin and triptorelin) than in other premenopausal women [91]. AIs could be classified into steroid (letrozole and anastrozole) and nonsteroid (exemestane and formestane) subtypes. Although the two subtypes interact with aromatase in different manners, their clinical efficacies are similar [92]. In contrast to SERMs and SERDs, estrogen deprivation leads to suppression of both ERα and ERβ signaling in target cells and affects immune regulation (see Table 1). Compared with ERα, ERβ is more dominantly expressed on immune cells, especially under inflammatory and hypoxic conditions [93], which may contribute differentially to the TIM under estrogen deprivation and SERMs/SERDs treatment (depicted in Fig. 2).

Multiple studies have demonstrated that AIs and/or GnRHa promote an antitumor TIM. For example, formestane treatment made ER + tumors more sensitive to ADCC by monocytes, suggesting a positive effect of AIs on antigen-specific antitumor immunity [94]. TH1 polarization in the T cell population was also induced by GnRHa, increasing the TNFα+/IL-10 + TH cell ratio [45]. In addition, Generali et al. and Chan et al. evaluated the changes in TIL subtypes in ER + BC patients before and after letrozole/exemestane treatment and found a reduction in Foxp3 + T cells and an increase in CD8 + T cell infiltration among AI responders [41, 95]. The abrogation of estrogen-induced Treg proliferation and immunosuppressive activity may be responsible for the increased CD8+/Treg ratio after AI treatment. Moreover, anastrozole administration in rat models inhibited the differentiation of naive CD4 + T cells into Tregs and reduced Treg counts in spleens and popliteal lymph nodes [42]. Additionally, recent studies revealed the importance of ERβ activation in the induction, maintenance and immunosuppressive activity of Foxp3 + Tregs [43, 96]. In this regard, estrogen withdrawal may be more effective in elevating the CD8+/Treg ratio in the TIM than SERMs or SERDs. Mast cells, as unique tissue-resident immune cells, are also involved in BC progression [97]. Rao et al. found that infiltrated mast cells within bladder cancer expressed higher levels of ERβ than non-infiltrated mast cells and further promoted tumor metastasis by enhancing ERβ/CCL-2/CCR-2/EMT/MMP-9 signaling in the TIM [44]. It is therefore reasonable to assume that the estrogen deprivation-elicited inhibition of ERβ signaling prevents the role of mast cells in promoting tumor invasion by decreasing CCL-2 expression. In summary, compared to SERMs and SERDs, estrogen deprivation by AIs or GnRHa abrogates both ERα and ERβ signaling in tumor and immune cells, exerting a strong effect in reshaping the TIM.

The association between the TIM and endocrine therapy response has been evaluated. Bioinformatic analyses based on gene expression data revealed a lower infiltration of M1 macrophages and a higher infiltration of Tregs and M2 macrophages within ER + tumors than ER-negative tumors, as well as an enrichment of anergic T cells in anastrozole-irresponsive ER + BC patients [98, 99]. Two studies implied that BC patients with high TIL numbers and baseline expression of immune-related genes, a subtype acknowledged to benefit from chemotherapy, responded poorly to anastrozole [100, 101]. Additionally, biomarker analysis of data from two clinical trials has evaluated the association between the TIM and neoadjuvant endocrine therapy (NET), yet different conclusions were drawn. The CARMINA-02 trial assessed 86 pre- and post-NET tumor samples, from patients treated with either anastrozole or fulvestrant, and found greatly increased TIL numbers in post-NET samples of responders but not in those of nonresponders [102]. In contrast, the DBCG trial revealed significantly increased TIL numbers in BC patients with poor response, who received letrozole as NET [103]. These discrepancies may be attributable to the different regimens used, suggesting the need for studies with more samples and corresponding pathological assessment. In addition, ER + BC cells grown under estrogen-free conditions presented an upregulation of PD-L1 as a result of ERα signaling abrogation [81], corroborating the possibility of combining NET with immunotherapy in ER + BC.

## The effects of inhibiting PI3K-AKT-mTOR pathway on the immune microenvironment

**Fig. 3:**
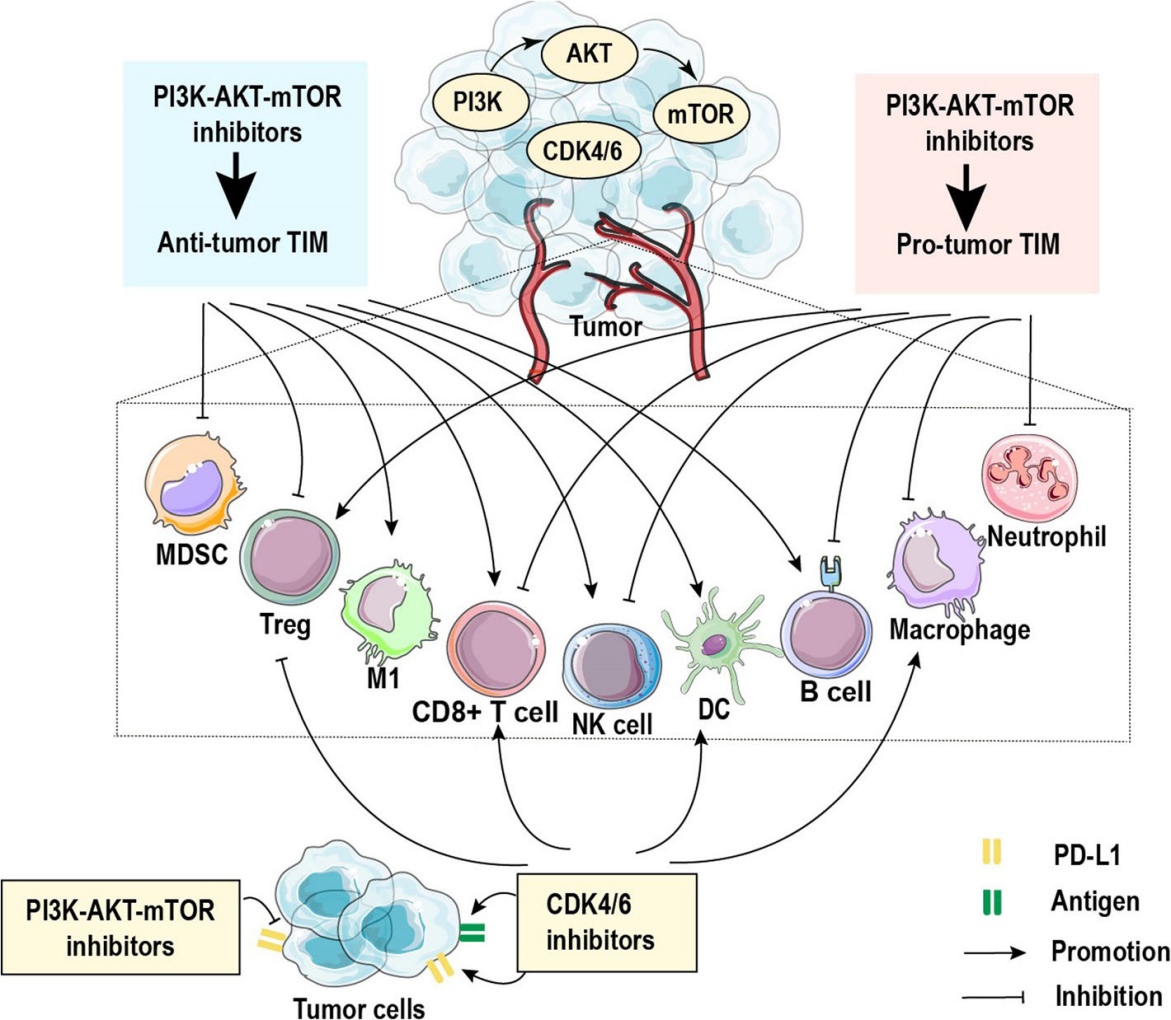
PI3K-AKT-mTOR inhibitors and CDK4/6 inhibitors orchestrate multiple effects on immune cells in the tumor immune microenvironment. The blue frame represents the antitumor immunity induced by PI3K-AKT-mTOR inhibitors. p110α and mTOR inhibitors decrease the number of MDSCs in tumors. The immunosuppressive function of MDSCs can be limited by p110δ and AKT inhibitors. p110δ inhibitors and long-term inhibition of mTOR also weaken Treg function. Moreover, p110γ and mTOR inhibitors promote the conversion of macrophages and mononuclear cells towards M1 phenotype. The function of CD8+ T cells is also enhanced by p110α, p110γ and p110δ inhibitors. p110α inhibitors suppress CD4+ T cells polarization towards Tregs, while pan-PI3K and AKT inhibitors are capable of selectively suppressing Treg proliferation. Additionally, pan-PI3K and p110γ inhibitors promote the tumor infiltration of T cells and B cells. Increased NK infiltration in tumors is also elicited by pan-PI3K inhibitors. Moreover, the function of DCs treated with mTOR inhibitors is significantly improved. The inhibition of PI3K-AKT-mTOR pathway also suppresses PD-L1 expression in BC cells, undermining PD-L1- mediated immunoresistance. The pink frame represents the protumor immunity induced by PI3K-AKT-mTOR inhibitors. pan-PI3K, p110β and mTOR inhibitors damage host defense mediated by macrophages, and p110β inhibition suppresses the cytotoxic function of neutrophils. p110δ inactivation shows an adverse effect on T cells and B cells. mTOR inhibitors can also suppress the effector function of T cells and NK cells, as well as promote Treg polarization. CDK4/6 inhibitors mainly induce antitumor immunity by improving the immunogenicity of BC cells and the antigen-presenting ability of DCs and macrophages. The CD8+/Treg ratio is also increased within tumors after CDK4/6 inhibitor treatment. The elevated level of PD-L1 in BC cells and immune cells mediated by CDK4/6 inhibitors supports the combined usage of CDK4/6 inhibitors with immune checkpoint inhibitor

PI3K-AKT-mTOR signaling is the most common aberrantly activated pathway in ER + BC, and this aberrant signaling has been acknowledged as the main cause of endocrine resistance [104]. Various targeted drugs for inhibiting this pathway have been developed to reverse endocrine resistance and have shown promising results [4]. In addition to staving tumor growth, inhibitors of this pathway also impact the functions of multiple immune cells (illustrated in Fig. 3). Therefore, the effects of these therapeutic strategies on the TIM deserve attention.

### PI3K inhibitors

PI3K inhibitors for ER + BC include pan-class I PI3K inhibitors targeting the p110α, p110β, p110γ and p110δ isoforms (buparlisib and pictilisib), as well as selective PI3K p110α inhibitors (alpelisib and taselisib) [4]. The results from the BELLE-2 study showed that the addition of buparlisib to fulvestrant prolonged progression-free survival (PFS) by 1.9 months among advanced ER + BC patients. However, the accompanying severe toxicity, including mental disorders, impaired liver function, hyperglycemia and rash, impeded its application [105]. Nevertheless, a nonsignificant improvement of PFS in ER + BC with a combination of pictilisib and fulvestrant was reported by the FERGI trial, and limiting the dosage of pictilisib due to toxicity might reduce its efficacy [106]. In contrast, preliminary clinical results showed a favorable clinical benefit and safety profile with selective p110α inhibitors plus fulvestrant [107, 108].

The four catalytic isoforms of class I PI3K are widely expressed across different cell types in mammals, while p110γ and p110δ are mainly expressed in leukocytes [109]. Numerous in vivo and in vitro studies have revealed the immunomodulatory effects of multiple types of PI3K inhibitors (see Table 1). Xie et al. reported that the pan-PI3K inhibitor LY blocked the production of proinflammatory cytokines from macrophages including IL-1β, IL-6, IL-8 and TNFα, and undermined macrophage motility [46]. Multiple pan-PI3K inhibitors caused impaired T cell function, as represented by decreased secretion of global cytokines and granzyme B, due to the inactivation of p110 isoforms β, γ and δ [48, 110]. These results suggest that the usage of pan-PI3K inhibitors may impair normal immune surveillance. On the other hand, in vivo and in vitro investigations suggested that selective inhibition of Treg proliferation and maintenance by pan-PI3K inhibitors was capable of enhancing the T cell response [47]. Treatment with the pan-PI3K inhibitor BKM120 in mice bearing breast tumors was found to increase the tumor infiltration of NK cells, B cells, and CD4 + and CD8 + T cells, favoring antitumor immunity [49]. Furthermore, an early study found a higher level of PD-L1 expression in breast tumor specimens with *PIK3CA* mutation or *PTEN* loss than in those with wild-type versions of these genes, and the pan-PI3K inhibitor wortmannin could attenuate PD-L1-elicited immunosuppression by blocking S6K1-mediated transcription of PD-L1 in BC cells with activated PI3K [111]. Considering the complex immunomodulatory effects of pan-PI3K inhibitors, the application of selective isoform inhibitors might be able to simultaneously eradicate tumor cells and avoid the immunosuppressive effects of pan-PI3K inhibitors.

p110α is emphasized as a key therapeutic target for ER + BC due to the high mutation frequency of its gene, *PIK3CA* (34.5%) [104]. The synergistic effect between a p110α-selective inhibitor and trastuzumab was reported in HER2 + BC mouse models [50]. Compared with a pan-PI3K inhibitor, the p110α-selective inhibitor preserved AKT activation in CD8 + TILs and showed a more potent effect in combination with the anti-neu antibody to increase the infiltration of CD8 + TILs. This may be because the important functions of p110γ and p110δ isoforms in T cells are suppressed by pan-PI3K inhibitors [50]. The importance of the p110α subunit in the T cell-dependent immune response was also illuminated by Aragoneses-Fenoll et al. [51], who reported that p110α^−/−^ CD4 + and CD8 + T cells elicited a potent effector function, including an increase in the production of cytokines, particularly IFNγ. In addition, elevated expression of lysosomal associated membrane protein 1 (LAMP-1) and granzyme B in p110α^−/−^ CD8 + T cells and diminished polarization of p110α^−/−^ CD4 + T cells towards Tregs were also found. The p110α deletion-induced improvement of antitumor immunity mediated by T cells delayed tumor progression in mice. Moreover, the p110α-selective inhibitor alpelisib combined with a CDK4/6 inhibitor was revealed to significantly improve tumor-infiltrating CD4 + and CD8 + T cell activity and reduce the abundance of MDSCs in TNBC mouse models [52].These findings strongly indicate a reshaping effect on the antitumor TIM induced by selective p110α inhibitor, as well as their potential, together with ICBs, in cancer therapy.

The importance of p110β in the development and progression of BC has been investigated [112]. Studies with athymic nude mice found a better efficacy of dual p110α/p110β inhibitors in ER + BC than of single inhibitors [113, 114], yet others have indicated a negative effect of p110β-specific inhibitors on immune cells. Leverrier et al. revealed that phagocytosis of mouse macrophages induced by apoptotic cells and FcγR can be depressed by anti-p110β antibodies, possibly due to the important role of p110β in G protein-coupled receptor and receptor tyrosine kinase signal transduction in macrophages [53]. p110β activity was required for neutrophil activation in response to adhesive surfaces and immune complexes. p110β inhibition could restrain the adhesion, spreading and ROS production of neutrophils, suppressing their phagocytic function [54]. Therefore, it is conceivable that pharmacological inhibition of p110β may counteract immune surveillance in BC.

In addition to the direct antitumor effect of selective p110γ inhibitors, they may have a positive effect on antitumor immune surveillance. Studies reported the capacity of pharmacological inhibition of p110γ in myeloid cells to restrict breast tumor inflammation and progression. Mechanistically, the activation of integrin α4β1 responsible for myeloid cell invasion into tumors was suppressed by p110γ inhibitor [55]. Selective inactivation of p110γ also promoted polarization of macrophages towards a more inflammatory M1 phenotype by stimulating NFκB activation and inhibiting C/EBPβ activation, leading to a restoration of CD8 + T cell function [56, 57]. Moreover, PI3Kγ^−/−^ mice with MMTV-PyMT tumors showed increased infiltration of antitumor leukocytes, including B cells and CD4 + and CD8 + T cells, into tumors, and these increased numbers of TILs with loss of PI3Kγ contributed to the diminution of tumor growth [49]. These results suggest that p110γ inhibition holds great potential to reshape the TIM. Intriguingly, the upregulated expression of PD-1 and CTLA-4 on infiltrated CD8 + T cells was also found in 4T1 tumor with p110γ inhibition [57]. Both p100γ blockade-induced antitumor immunity and upregulation of immune checkpoint expression support the combined usage of p110γ-selective inhibitor and ICBs, and the synergistic tumor-suppressing effects of such combinations have been demonstrated by multiple studies [49, 56, 57].

Similar to other isoforms, p110δ is widely expressed in leukocytes [109] and its expression level gradually increases as breast tumors progress [58]. A mouse model of TNBC under p110δ inhibitor IC87114 treatment exhibited tumor growth retardation as a result of the direct p110δ inactivation within tumor cells and macrophages accompanied by a reduction of tumor-infiltrating macrophages [58]. Moreover, administration of a p110δ inhibitor in 4T1 tumor-bearing mice undermined the function of Tregs and MDSCs, which disrupted tumor immune tolerance and further reignited CD8 + cytotoxic T cell-mediated tumor clearance [59]. Considering the positive effect of estrogen on the proliferation and function of Tregs and MDSCs, p110δ inhibitors may exhibit promising effects in ER + BC. However, a large body of data has also shown an adverse effect of p110δ on T cells and B cells, resulting in host immune deficiency. The p110δ inhibitor IC87114 suppressed IFNγ secretion by effector/memory T cells in both human and mice, weakening their effector responses [60]. Significant impairment in the proliferation, survival and differentiation of B cells was observed after exposure to a selective p110δ inhibitor [61]. Collectively, the dual effect of p110δ on immune cells calls for rigorous and comprehensive studies in the future to evaluate the therapeutic value of p110δ inhibitors in different malignant diseases and their impact on the TIM.

### AKT inhibitors

Protein kinase B (AKT), a key target of the PI3K pathway, possesses three isoforms named AKT1, AKT2 and AKT3. Hyperactivation of AKT is prevalent in ER + BC and relevant to endocrine resistance [115]. The FAKTION trial revealed that a pan-AKT inhibitor, capivasertib, improved PFS in AIs-resistant advanced ER + BC patients [7].

Similar to pan-PI3K inhibitor, administration of an AKT inhibitor to PTEN-deficient BC cell lines exerted a suppressive effect on PD-L1 expression that could be upregulated by the activation of PI3K-AKT-mTOR pathway, reversing the immunosuppression elicited by PD-L1 [111]. Selective depletion of Tregs within tumors was also induced by AKT inhibitors to further enhance antitumor immune responses and mitigate tumor growth [47]. Additionally, the importance of AKT in the differentiation of neoplastic MDSCs from myeloid precursors has been reported, and a specific AKT inhibitor hampered MDSC differentiation and viability [62]. However, the AKT1 and AKT2 isoforms play different roles in macrophage polarization. For example, AKT1 ablation in macrophages was reported to promote M1 phenotype polarization, which was attributed to its induction of miR-155 expression that targeted C/EBPβ, a master regulator of M2 differentiation, while AKT2 deletion resulted in an M2 phenotype [63]. Reports also suggested that the activation of AKT2 induces macrophage chemotaxis and BC cells metastasis [116, 117]. In this regard, in addition to inhibiting tumor cells, AKT inhibitors may contribute to the formation of an antitumor immune microenvironment to some extent (see Table 1).

### mTOR inhibitors

The mammalian target of rapamycin (mTOR), as a serine/threonine kinase in two complexes, mTORC1 and mTORC2, is a key regulators of cell growth, metabolism and autophagy. Everolimus, a rapamycin analog targeting mTORC1, has gained FDA approval for use in improving endocrine therapy resistance in advanced ER + BC [118]. Considering the role of mTOR in immune cell differentiation and function [119], the changes in the immune microenvironment caused by mTOR inhibitors deserve attention (see Table 1).

Indeed, increasing evidence has indicated the dual effects of mTOR inhibitors on the TIM. The secretion of IL-1β, IL-6, IL-8 and TNFα by macrophages involved in macrophage motility and adhesion was depressed by mTOR inhibitor AZD, thereby suppressing macrophage-mediated host defense [46]. mTOR signaling is also required for the development and activation of NK cells mediated by IL-15R signaling. mTOR inhibition with rapamycin led to impaired NK cell proliferation and reduced IFNγ and granzyme B production [64, 65]. Additionally, the impact of mTOR inhibition on Tregs has been investigated. Everolimus facilitated TGFβ-dependent Treg conversion from naive CD4 + T cells and Foxp3 stable expression by abating the activation of DNA methyl transferase 1 (DNMT-1) [70]. An anergic state of CD8 + T cells could also be induced by rapamycin: deficiency of mTORC1 activity among CD8 + T cells resulted in a low metabolic rate and increased longevity yet failed to differentiate memory T cells into effector cells, leading to a decreased cytotoxic function and anergic state. Meanwhile, inhibition of mTORC2 activity enhanced the generation of CD8 + memory T cells [67, 68, 120]. In mice bearing lung tumors, rapamycin administration impaired the recruitment of CD8 + T cells into the tumor accompanied by the ability of the vaccine to reduce infiltration of Tregs and MDSCs [69]. These results reveal that mTOR inhibitors are beneficial for tumor to escape immune surveillance.

However, emerging evidence has also suggested the potential for mTOR inhibitors to impose an antitumor immune environment. Beyond suppressing the immunoresistance mediated by PD-L1 through a similar mechanism as wortmannin [111], rapamycin was found to polarize mononuclear cells towards M1 macrophages, a phenotype less sensitive to the apoptotic effect of rapamycin [121, 122]. Rapamycin administration also impeded mouse M-MDSC differentiation and immunosuppressive function by restraining glycolysis and the iNOS pathway to reactivate antitumor immunity [123, 124]. Although mTOR inhibition can directly impair the cytotoxicity and chemotaxis of CD8 + T cells, myeloid DCs treated with rapamycin were found to have an increased expression of the costimulatory molecules CD40 and CD86 as well as a reduced expression of PD-L1, showing an enhanced ability to induce therapeutic CD8 + T cell responses [66, 125]. Rapamycin treatment also modulates the functional characteristics of γδT cells, which are important in BC and have received great attention [126]. The cytotoxicity of Vγ4γδ T cells was significantly boosted by rapamycin through increased expression of NKG2D and TNFγ [71]. In Vγ2Vδ2 T cells, mTOR inhibition led to an enhanced cytotoxicity and resistance to Fas-mediated apoptosis, as well as increased proliferation after antigen stimulation [72]. In addition, transient mTOR inhibition before TCR stimulation was revealed to reverse the hyporesponsiveness of Tregs, which was dependent on leptin-mTOR pathway, and promote the proliferation of functional Tregs [73]. However, chronic inhibition of mTOR eventually suppressed Treg expansion and even resulted in Treg anergy because the proliferation and suppressive ability of Tregs require high glycolytic metabolism, which is dependent of mTOR activity [73, 74]. These findings suggest a pleiotropic effect of mTOR inhibitors in BC, and implementing high-throughput techniques such as mass cytometry and single-cell RNA sequencing could provide more comprehensive data to evaluate the effect of such inhibitors on the TIM.

## The effects of CDK4/6 inhibitors on the immune microenvironment

The cyclin D/cyclin-dependent kinases 4 and 6 (CDK4/6)-retinoblastoma protein (RB) pathway holds a core position in the development of BC. CDK4/6 inhibitors (CDK4/6i), including abemaciclib, palbociclib and ribociclib, in combination with hormone therapy have been used to treat hormone receptor-positive (HR+), HER2-negative metastatic BC [127–129]. In addition to inducing tumor cell cycl e arrest, mounting evidence reveals the immune modulatory of CDK4/6i in the TIM of BC (depicted in Fig. 3 and Table 1).

CDK4/6i enhance the immunogenicity of BC cells through a variety of mechanisms. Abemaciclib and palbociclib boosted the production of type III IFNs of BC cells by abolishing the action of RB-E2F-DNMT1 axis, which further drove the expression of IFN-stimulated genes in an autocrine manner and enhanced tumor antigen presentation [77]. Abemaciclib monotherapy also upregulated the expression of MHC class I and II in tumor cells in favor of immune-mediated tumor clearance [76].

Administration of CDK4/6i inflames the TIM of BC by reversing “cold” tumors to “hot” tumor by repressing immunosuppressive cells and potentiating the infiltration and function of antitumor immune cells. Upon treatment with CDK4/6i, the abundance of tumor-infiltrating immunosuppressive myeloid cells was significantly reduced in breast tumors along with decreased levels of IL-6, IL-10, and IL-23, and these cytokines can depress TH1 response [75]. On the other hand, CDK4/6i enhanced antigen presenting capabilities of macrophages and DCs via upregulating MHC class I and II [76]. Observations disclosed that abemaciclib and palbociclib selectively inhibited the proliferation of Tregs but not CD8 + T cells, which may be attributable to the higher level of RB1 in Tregs [77]. Furthermore, the abundance of the TH1 chemokines CXCL-9 and CXCL-10 in the TIM was also boosted after CDK4/6i treatment, inducing the chemotaxis of T cells towards tumor sites [75]. Similarly, cell cycle arrest caused by CDK4/6i can also elicit the senescence-associated secretory phenotype (SASP), which features an increased richness of chemokines and inflammatory factors released from senescent cells, such as IL-1, IL-6 and IL-8. The SASP is supposed to promote the recruitment of immune cells, including T cells and macrophages [130, 131]. Beyond the effects of CDK4/6i in reshaping the CD8+/Treg ratio in the TIM, the function of T cells is directly enhanced by CDK4/6i. Palbociclib was revealed to enhance the nuclear translocation and activity of nuclear factor of activated T cells (NFAT) in T cells, which gave rise to T cell activation and IL-2 production mediated by NFAT signaling [75]. It is notable that NFAT governed transcriptional profiling associated with T cell exhaustion [132], but in the context of immunostimulation induced by CDK4/6i, NFAT mainly evoked the effector gene program. These results strongly support a positive contribution of CDK4/6i to inducing antitumor immunity.

Randomized clinical trials have indicated the efficiacy of ICBs to be related to PD-L1 expression in BC [133, 134]. In BC mouse models administered CDK4/6i, increased levels of PD-1 and CTLA-4 on infiltrating T cells were detected [52]. Wei et al. revealed that PD-L1 expression could be regulated by the cyclin D-CDK4-cullin 3- speckle-type POZ protein (SPOP) E3 ligase axis. Cyclin D-CDK4-mediated phosphorylation of SPOP was abrogated by CDK4/6i, promoting the degradation of SPOP in a proteasome-dependent manner and further reducing cullin 3-SPOP ubiquitin ligase-mediated PD-L1 degradation [135]. CDK4/6i induction of antitumor immunity and immune checkpoint expression in tumor cells and T cells strongly suggest the ability of CDK4/6i to skew “cold” type BC into “hot” tumor, implying their potential of being combined with ICBs [75–77, 135]. Clinical trials of abemaciclib plus pembrolizumab (NCT02779751) as well as the combination of letrozole, palbociclib and pembrolizumab (NCT02778685) for patients with ER + BC have been carried out (see Table 2) and results are to be expected in the future.

## Conclusions

HR + BC has been commonly considered a “cold” tumor. However, accumulating evidence has revealed a greatly increased mutation burden and dynamic changes of TIM in tumors after treatment, especially in advanced HR + BC [136, 137]. The anti-inflammatory function of estrogen is an important factor that shapes the immunosuppressive environment in malignant diseases, including BC. Therefore, most studies have revealed either anti-estrogen treatment with SERMs and SERDs or estrogen deprivation with AIs and GnRHa to boost the function of antitumor immune cells as well as decrease the abundance of immune suppressive cells.

In addition, the influence of small molecule inhibitors targeting the PI3K-AKT-mTOR pathway on the TIM has also been discussed here in our review. Despite the myriad of preclinical studies, controversial conclusions indicate that the real clinical impact of these regimens on TIM remain largely unclear. Recent studies have found that CDK4/6i, which inhibit the cell cycle, can inflame the TIM of HR + BC by repressing Tregs and increasing the infiltration and activation of antitumor immune cells within tumor. These results suggest the ability of CDK4/6i convert “cold” tumors to “hot” tumors, as well as their synergistic effect with ICBs in eradicating BC. However, most preclinical studies have been based on in vitro or mouse models, limiting their generalizability, and the potential therapeutic advantage of combining these agents with standard anti-estrogen treatments must be weighed against the risk of toxicities.

In the era of tumor immunotherapy, experimental strategies are under investigation to improve the efficacy of current anti-estrogen treatments and overcome endocrine therapy resistance. Multiple clinical trials (see Table 2) are currently underway to assess whether the combinations of ICBs with endocrine therapy could be a solution. According to the inclusion criteria, almost of these studies enrolled patients progressed on SERMs, SERDs or AIs, and NCT03280563 is also investigating the effect of atezolizumab plus fulvestrant in patients who have been resistant to CDK4/6 inhibitor, which is acknowledged as the most powerful regimen against ER + BC. Furthermore, we suggest that samples from previous clinical studies should be reassessed with high-throughput technologies, such as proteomics and single-cell approaches to illuminate the panoramic image of TIM by various treatments, which could be of great help in instructing the therapeutic strategy in HR + BC.

## Data Availability

Not applicable.

## Abbreviations

BC: Breast cancer
ERα: Estrogen receptor alpha
SERMs: Selective estrogen receptor modulators
SERDs: Selective estrogen receptor downregulators
AIs: Aromatase inhibitors: GnRHa: Gonadotropin-releasing hormone antagonists
TIM: Tumor immune microenvironment
NK cells: Natural killer cells
MMP-9: Matrix metalloproteinase-9
PI-9: Protease inhibitor
DCs: Dendritic cells
IDO: Indoleamine 2,3-dioxygenase
Tregs: Regulatory T cells
MDSCs: Marrow-derived immunosuppressive cells
PD-1: Programmed cell death1 receptor
IRF7: Interferon regulatory factor 7
IPSE: Immune polarizing side effects
PD-L1: Programmed death-ligand 1
TNBC: Triple negative breast cancer
ADCC: Antibody-dependent cytotoxicity
ICAM-1: Intercellular adhesion molecule-1
NETs: Neutrophil extracellular traps
PMA: Phorbol 12-myristate 13-acetate
TILs: High tumor infiltrating lymphocytes
NET: Neoadjuvant endocrine therapy
PFS: Progression free survival
LAMP-1: Lysosomal associated membrane protein 1
AKT: Protein kinase B
mTOR: The mammalian target of rapamycin
DNMT1: DNA methyl transferase 1
CDK4/6i: Cyclin D/cyclin-dependent kinases 4 and 6 inhibitors
RB: Retinoblastoma protein
HR: Hormone receptor
SASP: Senescence-associated secretory phenotype
NFAT: Nuclear factor of activated T cells
SPOP: Speckle-type POZ protein
